# Observational study on the efficacy and safety of erlotinib in patients with non-small cell lung cancer

**DOI:** 10.3892/ol.2012.1048

**Published:** 2012-11-27

**Authors:** TAKAYUKI KABURAGI, HIROAKI SATOH, KENJI HAYASHIHARA, TAKESHI ENDO, NOBUYUKI HIZAWA, KOICHI KURISHIMA, YOSHIHIRO NISHIMURA, TOSHIO HASHIMOTO, HIROYUKI NAKAMURA, KOJI KISHI, MASAHARU INAGAKI, TAKESHI NAWA, HIDEO ICHIMURA, HIROICHI ISHIKAWA, KATSUNORI KAGOHASHI, TOSHIHIKO FUKUOKA, YOKO SHINOHARA, KOICHI KAMIYAMA, YUKIO SATO, MITSUAKI SAKAI, TAKESHI MATSUMURA, KEIKO UCHIUMI, KINYA FURUKAWA

**Affiliations:** 1 Division of Respiratory Medicine, Ibaraki Prefectural Central Hospital and Regional Cancer Center, Kasama 309-1793;; 2 Mito Medical Center, University of Tsukuba, Mito 310-0015;; 3 Ibaraki Higashi Hospital, Tokai 319-1113;; 4 Mito Medical Center Hospital, Ibaraki 311-3193;; 5 University of Tsukuba Hospital, Tsukuba 305-8575;; 6 Mito Chuo Hospital, Mito 311-1135;; 7 Mito Saiseikai Hospital, Mito 311-4198;; 8 Tokyo Medical University Kasumigaura Hospital, Ami 300-0395;; 9 Tsuchiura Kyodo General Hospital and Regional Cancer Center, Tsuchiura 300-0053;; 10 Hitachi General Hospital, Hitachi 317-0077;; 11 Tsukuba Medical Center Hospital and Regional Cancer Center, Tsukuba 305-0005;; 12 JA Toride Medical Center, Toride 302-0022;; 13 Tsukuba Kinen Hospital, Tsukuba 300-2622;; 14 Ibaraki Seinan General Hospital, Sakai 306-0433, Japan

**Keywords:** erlotinib, non-small cell lung cancer, observational study, population-based

## Abstract

To evaluate the efficacy and safety of erlotinib for non-small cell lung cancer (NSCLC), we performed a population-based observational study. The study involved 307 patients treated with erlotinib at 14 sites (17 departments) in Ibaraki (Japan) between December 2007 and December 2010. The tumor response and disease control rates were 11.1 and 46.3% in all patients, respectively. The median time to treatment failure and survival time were 1.6 months (95% confidence interval, 41–57 days) and 5.3 months (134–181 days) in all patients, respectively. Survival was significantly prolonged in EGFR mutation-positive patients compared with negative patients. EGFR mutation-negative patients who presented with a skin rash had significantly prolonged survival compared with those without a skin rash. The most common adverse event was skin disorder, followed by diarrhea. Although 45.6% of the patients in this study received erlotinib as a fourth-line or subsequent treatment, the results from this study were similar to those of clinical studies. We deduce that erlotinib is effective against NSCLC and is tolerated in clinical practice.

## Introduction

Recently, in addition to conventional cytotoxic antitumor drugs, molecular-targeted drugs that target genes involved in the biological properties of cancer cells, such as growth, invasion, progression and metastasis, have been developed. Notably, the epidermal growth factor receptor (EGFR) has been demonstrated to be overexpressed in various types of cancer, including non-small-cell lung cancer (NSCLC), and to be involved in the growth of cancer cells. The therapeutic potential of gefitinib, which targets EGFR tyrosine kinase, was investigated in a placebo-controlled phase III study (ISEL) in patients with advanced or recurrent NSCLC who had received up to 2 regimens. Although the superiority of gefitinib relative to the placebo was not demonstrated (1), a subset analysis revealed that this drug significantly prolonged the survival of non-smokers and Asian individuals. These results were similar to those of a phase II clinical study conducted prior to the ISEL study (2). A prospective study has demonstrated that a greater antitumor effect of gefitinib was observed in patients with tumors carrying an EGFR mutation (3,4). In a phase III study (IPASS) of patients with previously untreated NSCLC, conducted mainly in Asian countries, the usefulness of gefitinib therapy was compared with that of carboplatin plus paclitaxel therapy. A subset analysis revealed that in EGFR mutation-positive patients, the gefitinib therapy significantly prolonged progression-free survival (PFS) as compared with the carboplatin plus paclitaxel therapy. By contrast, in patients who were EGFR mutation-negative, the carboplatin plus paclitaxel therapy was found to significantly prolong the PFS (5). Based on these results, gefitinib is considered to be less effective in EGFR mutation-negative patients.

Erlotinib, as with gefitinib, is an EGFR tyrosine kinase inhibitor (EGFR-TKI). In a phase III study (BR.21) that compared erlotinib with a placebo in the second- or third-line treatment of NSCLC patients who were not responding to standard chemotherapy, erlotinib was confirmed to significantly prolong overall survival, PFS and the time to deterioration of lung cancer-related symptoms (cough, dyspnea and pain) as a QOL measure (6). Successful results were also achieved in a combined analysis of two phase II clinical studies (JO16565 and JO18396) conducted in Japan. The objective response and disease control rates were 28% [95% confidence interval (CI), 20.0–37.9] and 49% (39.2–59.0), respectively, while the time to progression was 10.7 weeks (8.1–18.3) and the overall survival time was 13.8 months (11.4–18.1) (7). While erlotinib is demonstrated to be effective in EGFR mutation-positive patients (8,9), as is gefitinib, it is also suggested to be effective in EGFR mutation-negative patients (8,10).

However, the aforementioned findings were obtained in clinical studies; in clinical practice, a large number of patients who are excluded from clinical studies due to their characteristics are also treated. Recently, increasing numbers of advanced or recurrent NSCLC patients have received ≥2 regimens of chemotherapy due to the advances in chemotherapy, but adequate information is not available from clinical studies of the conventional second-line treatment. EGFR-TKIs in particular, which are orally administered, are frequently used for lung cancer treatment as their toxicity is typically low, although severe adverse reactions to the drug, including interstitial lung disease, have been demonstrated. However, studies investigating their efficacy and safety in Japanese individuals under conditions of actual use are limited (11). Hence, we set out to investigate the usefulness of erlotinib in lung cancer treatment by collecting and analyzing data from all patients receiving erlotinib, irrespective of their individual characteristics.

## Patients and methods

### Patients

Fourteen sites (17 departments) in Ibaraki (area, 6095 km^2^; population, ∼3 million) were involved in the current study, which included patients who were treated with erlotinib at these sites between December 2007 and December 2010. All patients demonstrated histological or cytological evidence of NSCLC. Histopathological diagnoses were defined by the World Health Organization (WHO) classification system, and patients were staged according to the Union for International Cancer Control (UICC) tumor node metastasis (TNM) staging system.

The patient characteristics, efficacy and safety were evaluated using patient data extracted from the database of each site. Tumor responses were classified as a complete response (CR), a partial response (PR), stable disease (SD), progressive disease (PD) or not evaluable (NE), according to the response evalution criteria in solid tumors (RECIST), version 1.1.

The present observational study conformed to the Ethical Guidelines for Clinical Studies issued by the Ministry of Health, Labor and Welfare of Japan.

### Statistical analysis

The patient survival time was calculated from the day of erlotinib therapy intitiation to the death or latest follow-up time of the patient. The survival rate was analyzed by the Kaplan-Meier method and comparisons were performed using the log-rank test. P<0.005 was considered to indicate a statistically significant difference.

## Results

### Patient characteristics

The final data set consisted of 307 patients. The patient characteristics are shown in Table I. The median age of the patients was 67 years (range, 32–91) and 34.2% of patients were female. Of the patients, 75.9% had adenocarcinoma, while the performance status (PS) was 0 or 1, 2, and 3 or 4 in 67.4, 20.5 and 12.1% of patients, respectively. In total, 17.9% of patients were EGFR mutation-positive and 27.7% were negative. Of the patients, 34.5% had never smoked and 63.2% had a history of smoking, while 70.4% received erlotinib as a third-line or subsequent treatment.

### Therapeutic effect

The tumor response rate was 11.1% (CR, 1.3%; PR, 9.8%) in the patients receiving erlotinib. SD was observed in 35.2% of patients and the disease control rate was 46.3%.

### Toxicity

The most common adverse event was skin disorder, which occurred in 177 patients (57.7%); 154 (50.2%) patients developed a grade 1 or 2 skin disorder. The second most common adverse event was diarrhea; occurring in 53 (17.3%) patients; 51 (16.6%) patients developed diarrhea of grade 1 or 2. Liver disorder was observed in 18 (5.9%) patients, none of which were grade 5. Interstitial lung disease was observed in 21 (6.8%) patients, two (0.7%) of whom developed an interstitial lung disease of grade 5.

### Survival analysis

**Figure 1.**
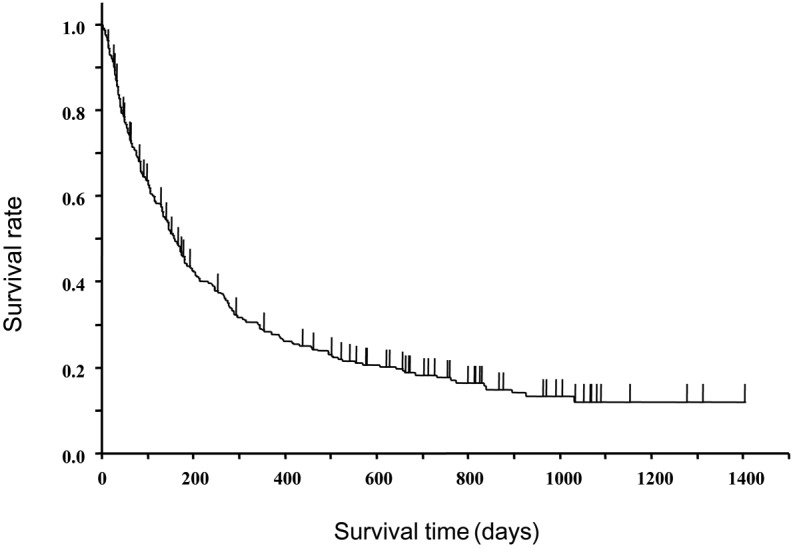
Survival curve of 307 patients with non-small cell lung cancer treated with erlotinib.

**Figure 2.**
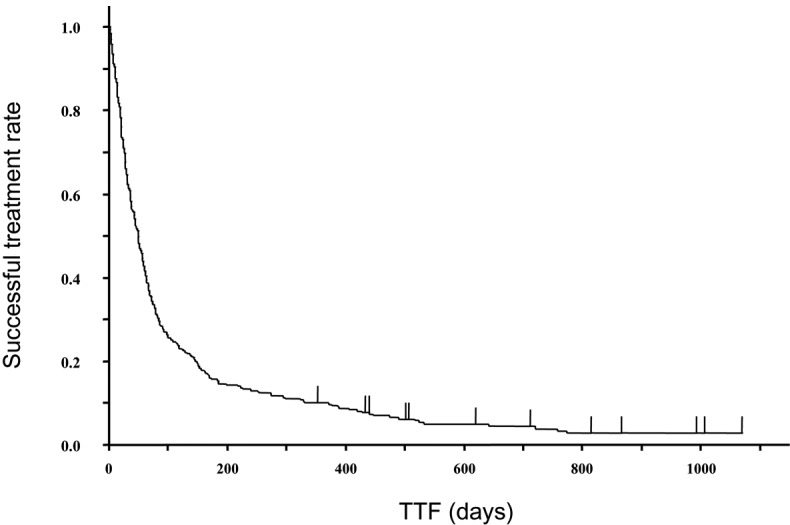
Time to treatment failure (TTF) of 307 patients with non-small cell lung cancer treated with erlotinib.

Of the 307 patients, 242 (78.8%) did not survive to the point of analysis. Figs. 1 and 2 show the survival curve and time to treatment failure (TTF) of the 307 patients treated with erlotinib. The median TTF and survival time were 1.6 months (95% CI, 41–57 days) and 5.3 months (134–181 days) in all patients, respectively. In male and female patients, the median TTFs were 1.5 months (95% CI, 36–56 days) and 1.7 months (40–68 days), and the median survival times were 4.9 months (127–180 days) and 5.8 months (131–270 days), respectively; no significant differences were observed in both of these measurements (P=0.05 and P=0.1008, respectively).

**Figure 3.**
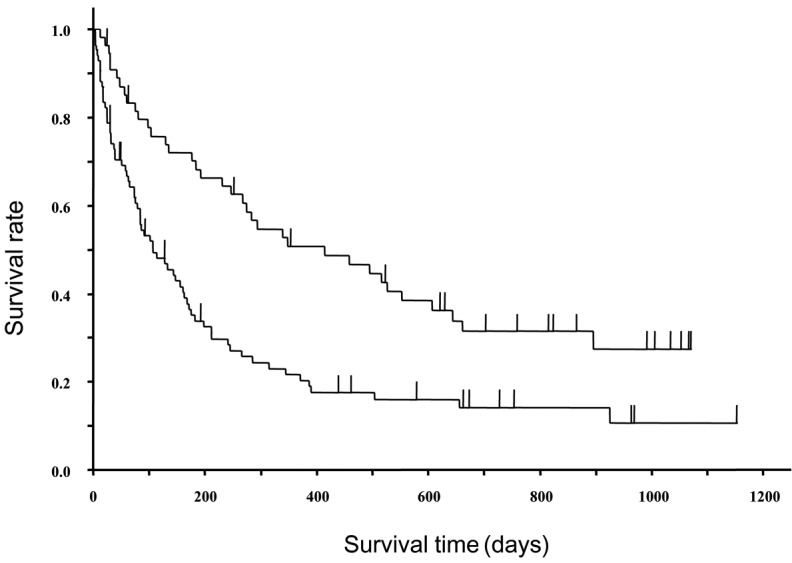
Survival curves of 55 EGFR mutation-positive (upper line) and 85 EGFR mutation-negative (lower line) patients. EGFR, epidermal growth factor receptor.

**Figure 4.**
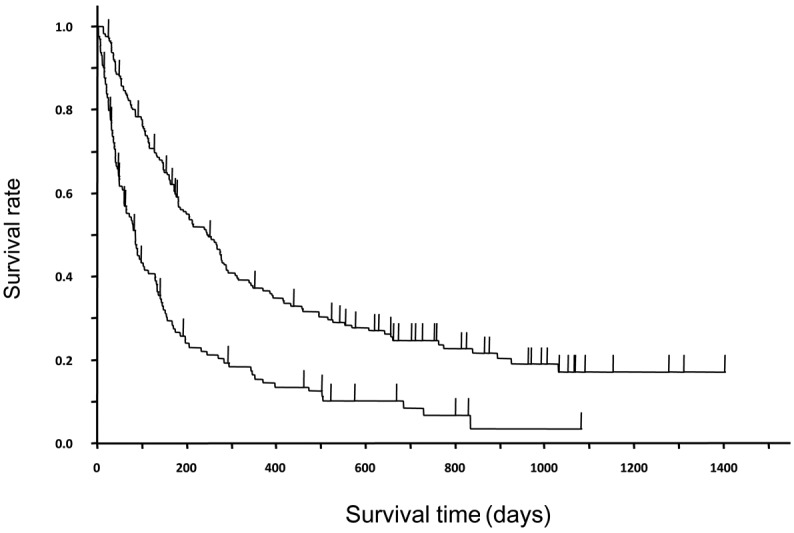
Survival curves of 130 patients with a skin rash (upper line) and 177 patients without a skin rash (lower line).

Fig. 3 shows the survival curves of EGFR mutation-positive and -negative patients. In EGFR mutation-positive and -negative patients, the median TTFs were 3.9 months (95% CI, 63–183 days) and 1.0 month (21–43 days), while the median survival times were 13.8 months (247–607 days) and 3.5 months (75–163 days), respectively; significant differences were observed in both of these characteristics (P<0.0001 and P=0.0002, respectively). In the EGFR mutation-positive patients, an earlier line of treatment typically resulted in a longer TTF; the TTFs were 721, 302, 142 and 73 days in the first-, second-, third- and fourth-line or subsequent treatments, respectively. However, in the EGFR mutation-negative patients, no significant differences were identified in TTF between the treatment lines; the TTFs were 34.5, 27, 40.5 and 29 days in the first-, second-, third- and fourth-line or subsequent treatments, respectively. In patients with and without a history of smoking, the median TTFs were 1.5 months (95% CI, 37–56 days) and 1.8 months (37–66 days), and the median survival times were 4.8 months (113–180 days) and 6.0 months (134–276 days), respectively. A significant difference was observed between survival times (P=0.0159). Fig. 4 shows the survival curves of patients with or without a skin rash as a complication of treatment with erlotinib. In patients with and without a rash, the median TTFs were 2.2 months (95% CI, 58–78 days) and 0.9 months (22–33 days), and the median survival times were 8.2 months (181–88 days) and 2.8 months (60–114 days), respectively. Significant differences were observed for both TTF and survival time between patients with and without a rash (P<0.0001 for both comparisons). The univariate analysis of the correlations between TTF and variables, including gender, age, treatment line, rash development and tumor response, showed that smoking history, PS, histological type, rash development and response to treatment were significant prognostic factors (Table II). The multivariate analysis of the correlation between survival and the aforementioned six factors showed that PS 0–1, rash development, at least a PR and at least SD were independent prognostic factors (Table III).

**Figure 5.**
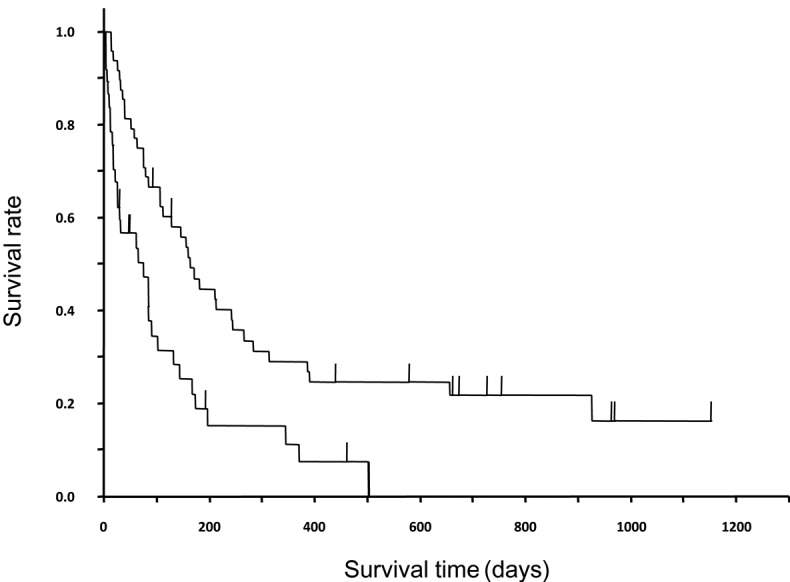
Survival curves of 48 EGFR mutation-negative patients with a skin rash (upper line) and 37 EGFR mutation-negative patients without skin rash (lower line). EGFR, epidermal growth factor receptor.

In the EGFR mutation-negative patients, there were significant differences in TTF (P=0.0003) and survival (P=0.0009) between those with and without a rash; the median values were 1.5 months (95% CI, 31–62 days) and 5.4 months (106–245 days) in the former, and 0.7 months (12–26 days) and 2.5 months (25–102 days) in the latter, respectively (Fig. 5).

## Discussion

In a phase III study (BR.21) that compared erlotinib with a placebo as the second- or third-line treatment of NSCLC patients who were not responding to standard chemotherapy, patients with a median age of 62 years receiving erlotinib had a median survival of 6.67 months and a median PFS of 2.2 months. In a phase III study (TITAN) that compared the efficacy of erlotinib with that of docetaxel or pemetrexed in the second-line treatment of NSCLC patients, the erlotinib group had a median overall survival of 5.3 months and a median PFS of 1.4 months (12). In the TITAN study, the median age of the erlotinib group (59 years) was lower than that of the patients in the present study. Additionally, only PS 0–2 patients were able to enrol, and 81% of the patients in the erlotinib group were PS 0–1 patients. In the present study, the median age of the patients was 67 years, the median survival time was of 5.3 months and the median TTF was 1.6 months; however, 45.6% of patients received erlotinib as a fourth-line or subsequent treatment. These results were similar to those of clinical studies where eligibility criteria for enrollment were established. As the present study has demonstrated a difference in the effect of erlotinib depending on the presence of a rash, we suggest that erlotinib may be more effective in patients with a rash than in those without a rash. Moreover, similar results were obtained in the EGFR mutation-negative patients, suggesting that erlotinib may be effective in EGFR mutation-negative patients, in whom EGFR-TKIs are considered to be less effective, on developing a rash. The incidence of adverse events in the present study was similar to that of post-marketing surveillance study of erlotinib in Japan; the rates of rash, liver disorder and diarrhea of grade 3 or more were 7.5, 1.6 and 0.7% in the former and 7.8, 1.8 and 1.23% in the latter, respectively. In contrast to the clinical studies that only included patients with a relatively good PS, the present study included all patients treated with erlotinib in clinical practice. We consider it important to investigate the efficacy and tolerability of erlotinib in this way.

## Figures and Tables

**Table I.** Characteristics of 307 patients.

Characteristic	Number	Percentage
Age (years)		
Median	67	
Range	32–91	
Gender		
Male	202	65.8
Female	105	34.2
Histological type		
Adenocarcinoma	233	75.9
Squamous cell carcinoma	42	13.7
Other	32	10.4
Tumor stage		
I–IIIA	23	7.5
IIIB	43	14.0
IV	177	57.7
Postoperative recurrence	64	20.8
PS		
0	82	26.7
1	125	40.7
2	63	20.5
3	30	9.8
4	7	2.3
Smoking history		
Non-smoker	106	34.5
Former smoker	178	58.0
Current smoker	16	5.2
Unknown	7	2.3
EGFR mutation		
Positive	55	17.9
Negative	85	27.7
Unknown	167	54.4
Metastatic focus		
Lung	121	39.4
Liver	48	15.6
Lymph node	124	40.4
Bone	105	34.2
Adrenal	6	2.0
Other	79	25.7
Treatment line		
First-line	20	6.5
Second-line	71	23.1
Third-line	76	24.8
Fourth-line or subsequent	140	45.6

PS, performance status; EGFR, epidermal growth factor receptor.

**Table II.** Univariate analysis for survival.

Characteristic	1	2	P-value^a^	Hazard ratio	95% CI
Smoking history	Yes	No	0.0147	0.717	0.544–0.937
PS	≥2	0/1	<0.0001	0.341	0.259–0.450
Histological type	Non-adenocarcinoma	Adenocarcinoma	<0.0001	0.548	0.414–0.733
Rash	No	Yes	<0.0001	0.459	0.355–0.595
RR	SD/PD/NE	CR/PR	<0.0001	0.199	0.109–0.331
DCR	PD/NE	CR/PR/SD	<0.0001	0.346	0.266–0.450

a P-value is 1 vs. 2. CI, confidence interval; PS, performance status; RR, response rate; DCR, disease control rate; SD, stable disease; PD, progressive disease; NE, not evaluable; CR, complete response; PR, partial response.

**Table III.** Multivariate analysis for survival.

Characteristic	1	2	P-value^a^	Hazard ratio	95% CI
PS	≥2	0/1	<0.0001	0.394	0.295–0.528
Rash	No	Yes	0.0002	0.596	0.455–0.782
RR	SD/PD/NE	CR/PR	<0.0001	0.328	0.176–0.565
DCR	PD/NE	CR/PR/SD	<0.0001	0.437	0.329–0.577

a P-value is 1 vs. 2. CI, confidence interval; PS, performance status; RR, response rate; DCR, disease control rate; SD, stable disease; PD, progressive disease; NE, not evaluable; CR, complete response; PR, partial response.
